# 5,6-Dichloro-2-Phenyl-Benzotriazoles: New Potent Inhibitors of Orthohantavirus

**DOI:** 10.3390/v12010122

**Published:** 2020-01-20

**Authors:** Giuseppina Sanna, Sandra Piras, Silvia Madeddu, Bernardetta Busonera, Boris Klempa, Paola Corona, Roberta Ibba, Gabriele Murineddu, Antonio Carta, Roberta Loddo

**Affiliations:** 1 Department of Biomedical Sciences, University of Cagliari, Cittadella Universitaria, 09042 Monserrato, Cagliari, Italy; silvia.madeddu@unica.it (S.M.); iurru99@hotmail.com (B.B.); rloddo@unica.it (R.L.); 2 Department of Chemistry and Pharmacy, University of Sassari, Via Muroni 23/A, 07100 Sassari, Italy; piras@uniss.it (S.P.); pcorona@uniss.it (P.C.); ribba@uniss.it (R.I.); muri@uniss.it (G.M.); 3 Institute of Virology, Biomedical Research Center Slovak Academy of Sciences, 845 05 Bratislava, Slovakia; boris.klempa@savba.sk; 4 Institute of Virology, Helmut-Ruska-Haus, Charité School of Medicine, 10117 Berlin, Germany

**Keywords:** orthohantavirus, phenyl-benzotriazoles, antiviral activity, C-FRA

## Abstract

Orthohantaviruses, previously known as hantaviruses (family Hantaviridae, order Bunyavirales), are emerging zoonoses hosted by different rodent and insectivore species. Orthohantaviruses are transmitted by aerosolized excreta (urine, saliva and feces) of their reservoir hosts. When transmitted to humans, they cause hemorrhagic fever with renal syndrome (HFRS) in Asia and Europe and hantavirus (cardio) pulmonary syndrome (HPS) in the Americas. Clinical studies have shown that early treatments of HFRS patients with ribavirin (RBV) improve prognosis. Nevertheless, there is the need for urgent development of specific antiviral drugs. In the search for new RNA virus inhibitors, we recently identified a series of variously substituted 5,6-dichloro-1(2)-phenyl-1(2)H-benzo[d][1,2,3]triazole derivatives active against the human respiratory syncytial virus (HRSV). Interestingly, several 2-phenyl-benzotriazoles resulted in fairly potent inhibitors of the Hantaan virus in a chemiluminescence focus reduction assay (C-FRA) showing an EC_50_ = 4–5 µM, ten-fold more active than ribavirin. Currently, there are no FDA approved drugs for the treatment of orthohantavirus infections. Antiviral activities and cytotoxicity profiles suggest that 5,6-dichloro-1(2)-phenyl-1(2)H-benzo[d][1,2,3]triazoles could be promising candidates for further investigation as a potential treatment of hantaviral diseases.

## 1. Introduction

Orthohantaviruses are classified as emerging viruses that cause two life-threatening diseases: hemorrhagic fever with renal syndrome (HFRS) and orthohantaviruses pulmonary syndrome (HPS), also known as hantavirus cardiopulmonary syndrome (HCPS) [1]. Small mammals are natural hosts of orthohantavirus, mainly rodents but recently reptiles and fishes [2] have also been discovered as carriers of these viruses that are transmitted to humans through the aerosol route. They are responsible for persistent infections without evident illness signs in their hosts [3]. The two diseases that are orthohantaviruses-related both induce an impressive rise in blood vessel permeability, strong immune responses and inflammation and viruses such as the Hantaan virus (HTNV) and Sin Nombre virus (SNV) are the causative agents. Although orthohantaviruses are distributed worldwide, HFRS and HCPS occur generally in Eurasia and the Americas, respectively [4].

Orthohantaviruses are members of the Hantaviridae family, order Bunyavirales. Their tripartite, single-stranded, negative sense RNA genome codes for four proteins. The L segment, S segment, and M segment encode an RNA-dependent RNA polymerase (RdRp), a nucleocapsid protein (N protein) and Gn and Gc glycoproteins, respectively. The two surface glycoproteins, before being exposed on the viral surface, are post-translationally processed via the endoplasmic reticulum and Golgi apparatus. These proteins interact with integrin receptors allowing viruses to enter new host cells [5].

The three genomic RNA molecules form a complex within the virion with N protein and, most probably, with RdRp. The viral RdRp mediates the genomic and anti-genomic viral RNAs and the transcription of viral mRNAs exclusively in the cytoplasm [6].

Over the last few years, the search for an effective treatment for orthohantaviruses infections has undergone a considerable increase [7]. Ribavirin (RBV), a broad-spectrum inhibitor, is the only antiviral with recognized in vitro and in vivo activity on hantavirus replication [8,9]. In China [10] and Russia [11], clinical trials have been conducted for the treatment of HFRS using post-exposure, intravenous RBV but while significant results have been obtained in the first case, the second resulted ineffective, as well as the trial conducted in patients with HPS. Furthermore, the use of RBV is limited by its myelosuppression and toxicity [12]. Besides RBV, the use of some nucleoside analogues resulted in being highly inhibitory in animal models [13]. A few other candidates have been evaluated: Favipiravir, a pyrazine derivative endowed of anti-influenza properties and Vandetanib, a tyrosine-kinase inhibitor. The use of corticosteroids, unfortunately, does not determine benefit and immunotherapy although it has given encouraging results; in preventing and treating human hantavirus infections, it remains challenging [14]. 

Nowadays, there are no U.S. Food and Drug Administration (FDA) granted antivirals [14], vaccines, or immunotherapeutic for the treatment of HFRS or HPS, and consequently, therapeutic approaches are usually based on supportive care. It is precisely for that reason that an effort to develop potential therapeutic agents is strongly desirable. At the present time, a very limited number of antivirals have been tested for orthohantavirus [14]. 

In recent years, our research group has published several 1(2)H-benzo[d][1,2,3]triazole, usually called benzotriazole, derivatives that have shown marked antiviral activity against many viruses [15,16,17,18]. The versatile biological behavior of benzotriazole and its derivatives have recently been described in an in-depth review [19]. Among these benzotriazole derivatives, the 5,6-dichloro1(2)phenyl-benzotriazole scaffold turned out to be endowed with high activity against several different viruses. 

**Figure 1:**
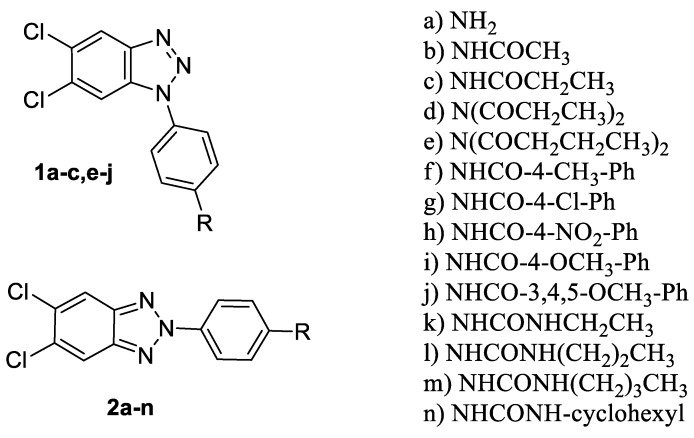
5,6-dichloro-1-phenyl-1*H*-benzo[*d*][1,2,3]triazoles (1a-c, e-j) and 5,6-dichloro-2-phenyl-2*H*-benzo[*d*][1,2,3]triazoles (2a-n).

In recent times, we described a series of 5,6-dichloro1(2)phenyl-benzotriazole derivatives that are active against te human respiratory syncytial virus (HRSV) in low micromolar range with low cytotoxicity (CC_50_ >100 µM) and very high selectivity when analyzed on a wide panel of positive- and negative-sense single-stranded RNA, double-stranded RNA, and DNA viruses [20]. In order to further investigate if these derivatives are able to inhibit viral infection processes of other negative sense RNA virus families, we subjected them (Figure 1) to a broad antiviral screening including the Hantaan virus (HTNV), a segmented RNA virus of the family Hantaviridae, for which there is currently no antiviral or approved vaccine. 

**Figure 2:**
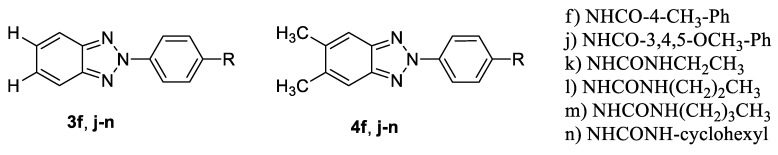
2-phenyl-2*H*-benzo[*d*][1,2,3]triazoles (3f, j-n) and 5,6-dimethyl-2-phenyl-2*H*-benzo[*d*][1,2,3]triazoles (4f, j-n).

In general terms, these molecules showed little or no activity except for HTNV. Series 2 derivatives (Figure 1) showed most interesting EC_50_ values, so we performed the same broad antiviral screening on a series of 2-phenyl-benzotriazole from our library (3k-n and 4k-n) [21] or newly synthetized (3f, j and 4f, j), all showed in Figure 2, in order to better understand the role of substituent in position C5 and C6 on the benzotriazole moiety and describe structure-activity relationships (SARs).

## 2. Materials and methods

### 2.1. Chemistry

5,6-dichloro-1-phenyl-1*H*-benzotriazoles (1a-c, e-j), 5,6-dichloro-2-phenyl-2*H*-benzotriazoles (2a-n), 1-(4-(2*H*-benzotriazol-2-yl)phenyl)-3-alkylureas (3k-n) and 1-(4-(5,6-dimethyl-2*H*-benzotriazol-2-yl)phenyl)-3-alkylureas (4k-n) were obtain as recently described [20,21].

### 2.2. General Procedure for Preparation of Derivatives 3f, j and 4f, j. 

**Scheme 1:**
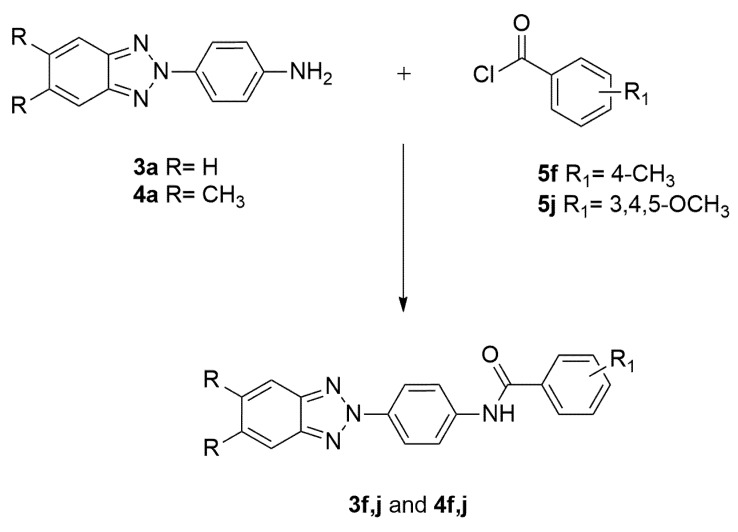
Synthesis the amide derivatives (3f, j and 4f, j).

Scheme 1 represents the synthetic route for obtaining derivatives 3f, j and 4f, j. 

To a stirred solution of 1.47 mmol of 4-(2*H*-benzo[*d*][1,2,3]triazol-2-yl)aniline (3a) or 4-(5,6-dimethyl-2*H*-benzo[*d*][1,2,3]triazol-2-yl)aniline (4a) in anhydrous *N*,*N*-dimethylformamide (8 mL), 4.98 mmol (ratio 1:3 for compound 3j) or 1.76 mmol (ratio 1:1 + 20% for compounds 3f, 4f, 4j) of proper benzoyl-chloride (5f, 5j) in anhydrous N,N-dimethylformamide (DMFa) were added. The mixture was stirred at 80 °C for 2 h (3j) or 24 h (3f, 4f, 4j). At the end, reaction mixture was cooled to room temperature and the solids were obtained by filtration. Mother liquor was dried by evaporation obtaining further solid. The crude solids were purified by flash chromatography, using a mixture of petroleum spirit/ethyl acetate in a ratio 7/3 as eluent, followed by crystallization from ethanol.

#### 2.2.1. *N*-(4-(2H-benzo[d][1,2,3]triazol-2-yl)phenyl)-4-methylbenzamide (3f)

Compound was obtained in 80% of total yield; m.p. 266–268 °C; TLC (petroleum spirit/ethyl acetate 1/1), Rf 0,90. ^1^H-NMR (DMSO-*d*_6_) δ: (1H, s, NHCO), 8.32 (2H, d, J = 8.4 Hz, H-2′, 6′), 8.09 (2H, d, J = 8.4 Hz, H-3′, 5′), 8.05-8.00 (2H, m, H-4, 7), 7.92 (2H, d, J = 7.6 Hz, H-2”, 6”), 7.53–7.50 (2H, m, H-5, 6), 7.37 (2H, d, J = 7.6 Hz, H-3”, 5”). ^13^C-NMR (DMSO-*d*_6_) δ: 165.60 (CO), 144.34 (C), 141.86 (2C), 140.36 (C), 134.90 (C), 131.72 (C), 128.94 (2CH), 127.77 (2CH), 127.43 (2CH), 120.89 (2CH), 120.68 (2CH), 118.04 (2CH), 21.00 (CH_3_). Anal. Calcd for C_20_H_16_N_4_O, C, 73.15; H, 4.91; N, 17.06. Found C, 73.23; H, 4.94; N, 16.98. MW 328.37, LC/MS: 329 (M + H).

#### 2.2.2. *N*-(4-(2H-benzo[d][1,2,3]triazol-2-yl)phenyl)-3,4,5-trimethoxybenzamide (3j)

Compound was obtained in 30% of total yield; m.p. 227–229 °C; TLC (petroleum spirit/ethyl acetate 7/3), Rf 0,29. ^1^H-NMR (DMSO-*d*_6_) δ: 10.44 (1H, s, NHCO), 8.34 (2H, d, J = 8.4 Hz, CH-2′, 6′), 8.04–8.00 (4H, m, CH-4, 7, 3′, 5′), 7.53–7.50 (2H, m, CH-5, 6), 7.33-7.28 (2H, m, CH-2”, 6”). ^13^C-NMR (DMSO-*d*_6_) δ: 165.17 (CO), 152.65 (2C), 144.35 (2C), 140.36 (C), 135.02 (C), 129.71 (2C), 127.48 (2CH), 121.15 (2CH), 120.72 (2CH), 118.03 (2CH), 105.41 (2CH), 60.12 (OCH_3_), 56.12 (2OCH_3_). Anal. Calcd for C_22_H_20_N_4_O_4_, C, 65.34; H, 4.98, N, 13.85. Found C, 65.46; H, 5.20; N, 13.58. MW 404.42, LC/MS: 405 (M + H).

#### 2.2.3. *N*-(4-(5,6-dimethyl-2H-benzo[d][1,2,3]triazol-2-yl)phenyl)-4-methylbenzamide (4f) 

This compound was obtained in 46% of total yield; m.p. 295–296 °C; TLC (petroleum spirit/ethyl acetate 7/3), Rf 0,53. ^1^H-NMR (DMSO-*d*_6_) δ: 10.46 (1H, s, NH), 8.25 (2H, d, J = 9.2 Hz, H-2′, 6′), 8.05 (2H, d, J = 9.2 Hz, H-3′, 5′), 7.91 (2H, d, J = 8.4 Hz, H-2”, 6”), 7.76 (2H, s, H-4, 7), 7.37 (2H, d, J = 8.4 Hz, H-3”, 5”), 2.51 (3H, s, CH_3_), 2.40 (6H, s, 2CH_3_). ^13^C-NMR (DMSO-*d*_6_) δ: 165.57 (CO), 143.79 (2C), 141.85 (C), 139.88 (C), 137.86 (2C), 135.06 (C), 131.73 (C), 128.95 (2CH), 127.74 (2CH), 121.40 (2CH), 120.90 (2CH), 116.17 (2CH), 29.96 (CH_3_), 20.99 (2CH_3_). Anal. Calcd for C_22_H_20_N_4_O, C, 74.14; H, 5.66; N, 15.72. Found C, 74.18; H, 5.82; N, 15.67. MW 356.42; LC/MS: 457 (M + H).

#### 2.2.4. *N*-(4-(5,6-dimethyl-2H-benzo[d][1,2,3]triazol-2-yl)phenyl)-3,4,5-trimethoxybenzamide (4j) 

This compound was obtained in 20% of total yield; m.p. 272–273 °C; TLC (petroleum spirit/ethyl acetate 6/4), Rf 0,34. ^1^H-NMR (DMSO-*d*_6_) δ: 10.40 (1H, s, NH), 8.28 (2H, d, J = 8.8 Hz, CH-2′, 6′), 8.02 (2H, d, J = 9.2 Hz, CH-3′, 5′), 7.76 (2H, s, CH-2”, 6”), 7.32 (2H, s, CH-4, 7), 3.90 (6H, s, 2OCH_3_), 3.75 (3H, s, OCH_3_), 2.41 (6H, s, 2CH_3_). ^13^C-NMR (DMSO-*d*_6_) δ: 165.11 (CO), 152.64 (2C), 143.8 (2C), 140.47 (C), 139.68 (C), 137.88 (2C), 135.18 (C), 129.73 (C), 121.14 (2CH), 120.32 (2CH), 116.26 (2CH), 105.38 (2CH), 60.12 (OCH_3_), 56.12 (2OCH_3_), 20.41 (2CH_3_). Anal. Calcd for C_24_H_24_N_4_O_4_, C, 66.65; H, 5.59; N, 12.96. Found C, 69.46; H, 5.82; N, 12.58. MW 432.47; LC/MS: 433 (M + H).

### 2.3. Cells, Viruses, Reagents

All experimental work involving viruses was performed in a biosafety level 3 (BSL3) containment laboratory. Hantaan (HTNV) (strain 76–118) (kindly provided by Prof. Dr. D. H. Krüger and Dr. Boris Klempa, Institute of Virology, Charitè University of Berlin) was used for all experiments. Vero E6 cells (ATCC CRL 1586) were maintained in complete EMEM (minimum essential medium with Earle’s salt, 25mM Hepes supplemented with 10% fetal bovine serum, 1% glutamine, 1% sodium pyruvate (NaPy), 1% non-essential amino acids NEAA, 0.1% gentamicyn). RBV was purchased from Sigma–Aldrich Co. (St. Louis, MO, USA). The primary antibody, rabbit anti-Malacky Ab, was obtained from Dr. Boris Klempa, Berlin. The secondary antibody was goat anti-rabbit IgG (H+L) HRP conjugate (Cat. No. 170-6515, Bio-Rad, Inc., CA, USA). SuperSignal West Pico Chemiluminescent Substrate (Cat. No. 34080, Thermo Scientific, MA, USA)

### 2.4. Cytotoxicity Assays 

Vero E6 cells were seeded at an initial density of 4 × 10^5^ cells/mL in 6-well plates, in culture medium (EMEM 25mM HEPES buffer) supplemented with 1% L-glutamine, 10% fetal bovine serum (FBS), 1% NaPy, 1% NEAA, and 0.1% gentamycin. Cell cultures were then incubated at 37 °C in a humidified, 5% CO_2_ atmosphere in the absence or presence of serial dilutions of test compounds. Cell viability was determined after 7 days at 37 °C by the crystal violet staining method.

### 2.5. Orthohantavirus Antiviral Screening Assay

Vero-E6 cells were seeded in 6-well plates at an initial density of 4 × 10^5^ cells/mL. Cell cultures were then infected and incubated at 37 °C in a humidified, 5% CO_2_ atmosphere for 7 days in the absence or presence of serial dilutions of test compounds. The antiviral activity was determined by chemiluminescence focus reduction assay (C-FRA), as described previously [22] with small modifications. Briefly, after 7 days of incubation, overlay medium were discarded and cell monolayers were fixed with ice-cold methanol for 8 min at room temperature. After fixing, the methanol was removed, and the cells let dry. Cells were rinsed twice with washing buffer, and then a rabbit anti-Malacky antibody was added at 1:1000 dilution for 1 h at 37 °C in a humidified, 5% CO_2_ atmosphere. Cells were then washed five times with washing buffer and a secondary goat anti-rabbit IgG antibody conjugated to horseradish peroxidase (HRP) was added for an additional hour in 5% CO_2_ atmosphere. After washing five times with washing buffer cells were incubated with chemiluminescent substrate immediately before the detection. Infected cell foci were detected with the CCD camera for 3 min (or Chemidoc for 30 sec).

### 2.6. Yield Reduction assay

Vero E6 cells were inoculated with HTNV at an m.o.i. of 0.05 in maintenance medium and tested compounds at non-cytotoxic concentrations. Following a 1-h adsorption period at 37 °C and 5% CO_2_ on a rocking platform, the inoculum was removed and replaced with fresh medium containing 20 µM concentration of compounds (2j, 2l, 2n). After 3 days, the cell supernatant was harvested and centrifuged (at 3000 rpm, at 4 °C, for 10 min) to remove debris and measured for the presence of infectious orthohantavirus hantavirus. The infectious progeny virus in the cell supernatant was evaluated by chemiluminescence focus reduction assay, as described above [22]. RBV was used as the reference compound.

### 2.7. Statistical Analysis

Data are represented as mean ± standard deviation (SD) unless otherwise stated. For the yield reduction assay, statistical comparisons were performed using the unpaired *t*-test, and *p*-values less than 0.05 were considered to be statistically significant. All analyses were performed with GraphPad Prism 6. 

## 3. Results and Discussion

**Figure 3:**
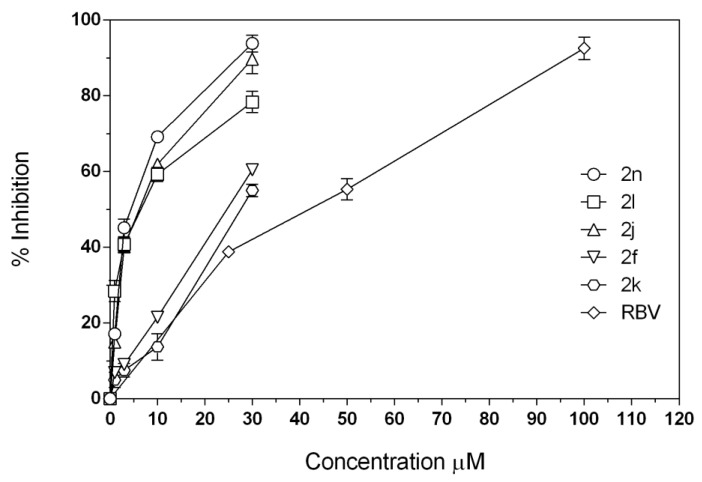
Anti-hantaviral effect of 2n, 2l, 2j, 2f, 2k, and RBV in vitro. Each symbol shows mean values for three independent determinations + SD.

In Figure 3, we report the effects of compounds 5,6-dichloro-2-phenyl-2H-benzo[d][1,2,3]triazoles (2n, 2l, 2j, 2f, 2k) on the replication of HTNV in comparison to RBV, a known broad-spectrum antiviral agent. Series 1, 3, and 4 derivatives turned out as completely inactive and are not reported here (see Table S1). 

Compounds 1a-c, 1e-j, 2a-n, 3f, 3j-n, and 4f, 4j-n, were evaluated for their potential inhibitory activity against HTNV using a chemiluminescence focus reduction assay (C-FRA). Three compounds (2j, 2l, 2n) showed interesting inhibitory activity (i.e., ≤5 µM; Figure 3). In parallel, moderate to low cytotoxicity was detected for almost all compounds, with CC_50_ values mostly in the high micromolar range (>30 µM) against all tested cell lines (see Table S1).

From a structure–activity relationship perspective, the most relevant results concerned the potent and selective activity of two urea derivatives (2l and 2n) and one amide derivative (2j), all belonging to the series of the 5,6-dichloro-2-phenyl-derivatives. By contrast, none of the molecules of the series 5,6-dichloro-1-phenyl-derivatives exhibited anti-HTNV activity, with only the exception of the compound 1h (EC_50_ = 21 µM). Regarding the presence of the two chlorine atoms in positions C5 and C6 of benzotriazole, their elimination (3f, j-n) or substitution with methyl groups (4f, j-n) results in total loss of antiviral activity. Moreover, we can highlight that derivative 2l (R= NHCONH-propyl) showed an EC_50_ value of 5 µM, but when decreasing or increasing the steric hindrance of the side chain with ethyl-group (2k) or butyl-group (2m), it turned out in a total loss of antiviral activity. 

**Figure 4:**
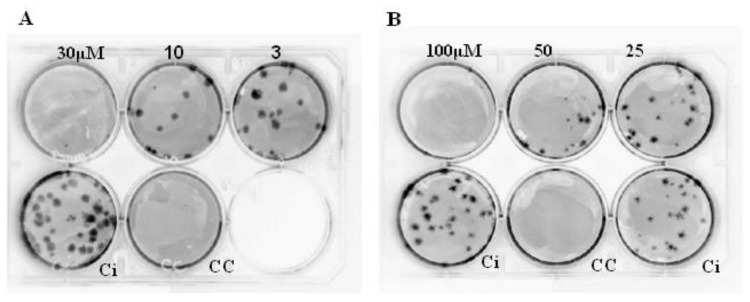
Focus reduction assay for 2n and ribavirin.VeroE6 cells were infected with HTNV (m.o.i. 0.05). The infected cultures were treated with 2n (panel **A**) and RBV (panel **B**; used as reference) at indicated doses. Ci = untreated control; CC = uninfected control. Antiviral activity was determined by focus reduction assay at day 7 post-infection.

Derivative 2n, recently described by our research group as an interesting inhibitor of HRSV entry [16], showed a remarkable EC_50_ value of 4 µM against HTNV, resulting in being ten-fold more potent than RBV (EC_50_ = 37 µM); Figure 4.

2n also resulted in being more active in in vitro assays against HTNV than two orthohantavirus candidate drugs: ETAR (1-beta-d-ribofuranosyl-3-ethynyl-[1,2,4] triazole) (EC_50_ = 10 µM) and Favipiravir (EC_50_ =150.8 μM), respectively 2- and 37-fold more potent [13,23].

**Figure 5:**
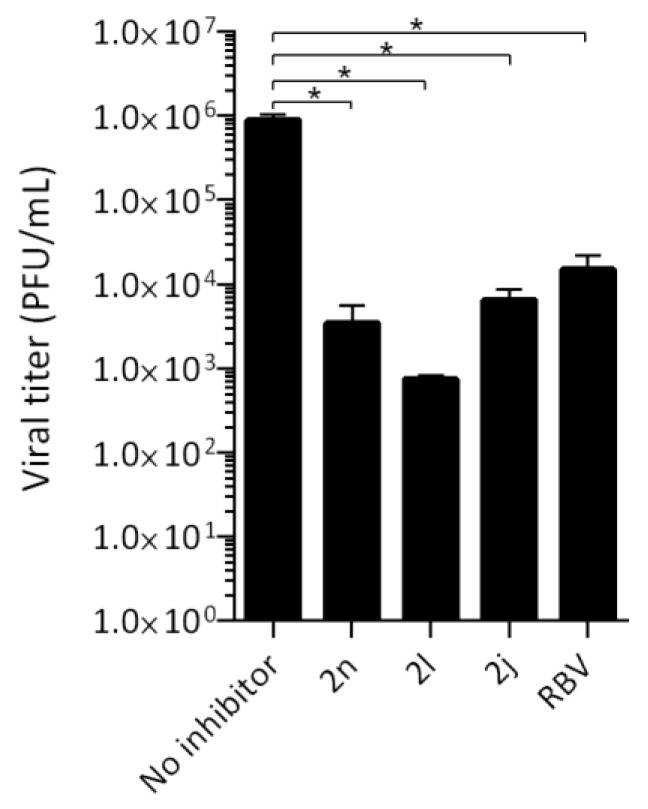
Yield of infectious Hantaan (HTNV) viruses produced in infected VeroE6 cells treated with selected benzotriazoles (2j, 2l, 2n) (20 µM) and RBV (50 µM). Results are expressed as means ± standard deviations from 3 separate experiments done in triplicate. * indicates the *p*-value <0.05.

Accordingly, to obtain a detailed insight on the efficacy of 2j, 2l, and 2n against HTNV, a yield reduction assay was performed. The reduction of virus titer in the presence of the active compound during a single round of viral infection was determined. Treatment with non-cytotoxic 20 µM concentration of 2j, 2l and 2n caused a significant reduction of viral titer (Figure 5). A significant decline in viral titer of HTNV was also observed at 50 µM treatment of RBV (* *p*-value <0.05, unpaired *t*-test).

VeroE6 cells were infected with HTNV (m.o.i. 0.05). The infected cultures were treated with 2j, 2l, 2n at indicated doses, and RBV was used as a reference compound. Viral yields in the culture supernatant were determined by viral titer reduction assay at day 3 post-infection. 

In our assay, benzotriazole derivatives 2n, 2l, 2j (20 µM) determined a very interesting reduction of viral titer compared to control cells infected in the absence of inhibitors. The production of the virus was significantly reduced upon treatment with 2l (3,08 log10). The same trend of reduction in viral loads was detected for 2n (2,41 log10) and 2j (2.14 log10). Conversely, in order to obtain a titer decrease, less than 2 logs (1,78 log10) if compared to untreated control, the reference molecule ribavirin needed to be added at 50 µM concentration. 

## 4. Conclusions

Over the past 50 years, hantavirus infections have caused severe diseases with serious clinical effects on human health. Recently, this has become more evident with the emergence of new outbreaks. As mentioned, there are no FDA approved antivirals, vaccines, or immunotherapeutic agents available for the treatment of HFRS or HPS. The objective of this study was to evaluate the antiviral activity of 2-phenyl-benzotriazoles against HTNV.

All compounds were generally endowed with low cytotoxicity against cell monolayers employed in our assays to support the replication of HTNV but also against a panel of cell cultures, as reported in SM2. We identified three promising lead compounds, derivatives 2j, 2l and 2n, characterized by interesting inhibitory activity against HTNV, in vitro ten-fold more potent than the nucleoside analogue RBV, currently the only antiviral with recognized in vitro and in vivo activity. This is the first report detailing benzotriazole anti-hantavirus activity. In conclusion, the therapeutic potential of these derivatives could be considered as a good starting point for the development of second-generation effective candidates for treatment against orthohantavirus infections. 

## Supplementary Materials

The following are available online at https://www.mdpi.com/1999-4915/12/1/122/s1, Table S1: Cytotoxicity and antiviral activity of phenyl-benzotriazoles against HTNV.
