# Supplementary material for: 5,6-Dichloro-2-Phenyl-Benzotriazoles: New Potent Inhibitors of Orthohantavirus
Source: Viruses. 2020 Jan 20;12(1):122. doi: 10.3390/v12010122 (PMC7019903; doi:10.3390/v12010122)
Supplement: Supplementary file 1 [file viruses-12-00122-s001.pdf]

Table S1. Cytotoxicity and antiviral activity of phenyl-benzotriazoles against HTNV

**Series 1** compounds	R	Vero E6	HTNV	Vero-76	MT-4	MDBK	BHK-21
		CC_{50}[a]	EC_{50}[b]	CC_{50}[c]			
**a**	NH_2	>30	>30	>100	≥100	>100	>100
**b**	$NHCOCH_3$	20	>20	30	35	43	53
**c**	$NHCOCH_2CH_3$	20	>20	30	28	≥100	54
**e**	$N(COCH_2CH_2CH_3)_2$	25	>25	20	35	14	16
**f**	NHCO-4-CH_3-Ph	>30	>30	>100	>100	>100	≥100
**g**	NHCO-4-Cl-Ph	>30	≥30	≥100	>100	>100	>100
**h**	NHCO-4-NO_2-Ph	>30	**21**	>100	>100	>100	96
**i**	NHCO-4-OCH_3-Ph	>30	>30	>100	33	>100	>100
**j**	NHCO-3,4,5-OCH_3-Ph	>30	>30	90	77	>100	>100
**Series 2** compounds							
**a**	NH_2	>30	>30	≥100	52	≥100	>100
**b**	$NHCOCH_3$	>30	>30	>100	>100	>100	>100
**c**	$NHCOCH_2CH_3$	>30	>30	>100	>100	>100	>100
**d**	$N(COCH_2CH_3)_2$	>30	>30	≥100	15	73	26
**e**	$N(COCH_2CH_2CH_3)_2$	>30	>30	≥100	24	84	62
**f**	NHCO-4-CH_3-Ph	>30	**22**	>100	>100	>100	>100
**g**	NHCO-4-Cl-Ph	>30	>30	95	>100	>100	>100
**h**	NHCO-4-NO_2-Ph	>30	>30	>100	>100	>100	>100
**i**	NHCO-4-OCH_3-Ph	>30	>30	>100	≥100	>100	>100
**j**	NHCO-3,4,5-OCH_3-Ph	>30	**4** (>7.5)	80	63	>100	35
**k**	$NHCONHCH_2CH_3$	>30	**26**	>100	>100	>100	>100
**l**	$NHCONH(CH_2)_2CH_3$	30	**5** (6)	30	>100	78	40
**m**	$NHCONH(CH_2)_3CH_3$	>30	>30	95	>100	>100	>100
**n**	NHCONH-cyclohexyl	>30	**4** (>7.5)	90	>100	>100	71
**Series 3** compounds							
**f**	NHCO-4-CH_3-Ph	>30	>30	>100	>100	>100	>100
**j**	NHCO-3,4,5-OCH_3-Ph	>30	>30	>100	>100	>100	>100
**k**	$NHCONHCH_2CH_3$	>30	>30	>100	nd	>100	>100
**l**	$NHCONH(CH_2)_2CH_3$	>30	>30	>100	>100	>100	>100
**m**	$NHCONH(CH_2)_3CH_3$	>30	>30	>100	>100	>100	>100
**n**	NHCONH-cyclohexyl	>30	>30	95	>100	>100	>100
**Series 4** compounds							
**f**	NHCO-4-CH_3-Ph	>30	>30	>100	>100	>100	>100
**j**	NHCO-3,4,5-OCH_3-Ph	>30	>30	>100	nd	>100	>100
**k**	$NHCONHCH_2CH_3$	>30	>30	>100	>100	>100	>100
**l**	$NHCONH(CH_2)_2CH_3$	>30	>30	>100	>100	>100	>100
**m**	$NHCONH(CH_2)_3CH_3$	>30	>30	>100	>100	>100	>100
**n**	NHCONH-cyclohexyl	>30	>30	95	>100	>100	>100
**Reference** compound							
Ribavirin RBV		>100	37 (>2)				

Data represent mean values + SD for three independent determinations. For values where SD is not shown, variation among triplicate samples was less than 15%. Results for active compounds are in bold character.

[a]Compound concentration (μM) affecting the morphology of Vero-E6 monolayers, as determined by optical microscope examination.

[b]Compound concentration (μM) required to reduce the foci number of HTNV by 50% in Vero E6 monolayers.

[c]Compound concentration (μM) required to reduce the viability of mock-infected MT-4, Vero-76, MDBK and BHK21 cells by 50%, as determined by the MTT method.

( ) Selectivity Index

Cells

Cell lines were purchased from American Type Culture Collection (ATCC): Vero E6 cells (ATCC CRL 1586) [B. Klempa, P. T. Witkowski, E. Popugaeva, B. Auste, L. Koivogui, E. Fichet-Calvet, T. Strecker, J. ter Meulen, D. H. Krüger, Sangassou Virus, the First Hantavirus Isolate from Africa, Displays Genetic and Functional Properties Distinct from Those of Other Murinae-Associated Hantaviruses. J Virol. (2012); 86(7): 3819–3827]; CD4+ human T-cells containing an integrated HTLV-1 genome (MT-4) ; Madin Darby Bovine Kidney (MDBK) [ATCC CCL 22 (NBL-1) Bos Taurus]; Baby Hamster Kidney (BHK-21) [ATCC CCL 10 (C-13) Mesocricetus auratus]; Monkey kidney (Vero-76) [ATCC CRL 1587 Cercopithecus Aethiops] [G. Sanna, S. Madeddu, G. Giliberti, S. Piras, M. Struga, M. Wrzosek, G. Kubiak-Tomaszewska, A.E Koziol,. O. Savchenko, T. Lis, J. Stefanska, P. Tomaszewski, M. Skrzycki, D. Szulczyk, Synthesis and Biological Evaluation of Novel Indole-Derived Thioureas. *Molecules* (2018), *23*, 2554].

Cytotoxicity assays

Exponentially growing MT-4 cells were seeded at an initial density of $4x10^5$ cells/ml in 96-well plates in RPMI-1640 medium, supplemented with 10% fetal bovine serum (FBS), 100 units/mL penicillin G and 100 μg/mL streptomycin. MDBK, BHK were seeded in 96-well plates at an initial density of $6x10^5$ and $1x10^6$, respectively, in Minimum Essential Medium with Earle's salts (MEM-E), L-glutamine, 1mM sodium pyruvate and 25 mg/L kanamycin, supplemented with 10% horse serum (MDBK) or 10% fetal bovine serum (FBS) (BHK-21). Vero-76 cells were seeded in 96-well plates at an initial density of $5x10^5$ cells/mL, in Dulbecco's Modified Eagle Medium (D-MEM) with L-glutamine and 25 mg/L kanamycin, supplemented with 10% FBS. Cell cultures were then incubated at 37 °C in a humidified, 5% CO_2 atmosphere, in the absence or presence of serial dilutions of test compounds. The test medium used for the cytotoxic assay as well as for antiviral assay contained 1% of the appropriate serum. Cell viability was determined after 72-120 hrs at 37 °C by MTT method [R. Pauwels, J. Balzarini, M. Baba, R. Snoeck, D. Schols, P. Herdewijn, J. Desmyter, E. De Clercq, Rapid and automated tetrazolium-based colorimetric assay for the detection of anti-HIV compounds, J. Virol. Methods 20 (1988) 309-321]. Vero E6 cells were seeded at an initial density of $4x\ 10^5$ cells/mL in 6-well plates, in culture medium (EMEM 25mM HEPES buffer) supplemented with 1% L-glutamine, 10% fetal bovine serum (FBS), 1% sodium pyruvate (NaPy), 1% non-essential amino acids (NEAA) and 0.1% gentamycin. Cell cultures were then incubated at 37 °C in a humidified, 5% CO_2 atmosphere in the absence or presence of serial dilutions of test compounds. Cell viability was determined after 7 days at 37 °C by the Crystal violet staining method.
